# Therapeutic beta-lactam dosages and broad-spectrum antibiotics are associated with reductions in microbial richness and diversity in persons with cystic fibrosis

**DOI:** 10.1038/s41598-023-27628-x

**Published:** 2023-01-21

**Authors:** Andrea Hahn, Aszia Burrell, Hollis Chaney, Iman Sami, Anastassios C. Koumbourlis, Robert J. Freishtat, Keith A. Crandall, Edith T. Zemanick

**Affiliations:** 1 Division of Infectious Diseases, Children’s National Hospital (CNH), Washington, DC USA; 2 Center for Genetic Medicine Research, Children’s National Research Institute, Washington, DC USA; 3 Department of Pediatrics, George Washington University (GWU), Washington, DC USA; 4 Division of Pulmonary Medicine, CNH, Washington, DC USA; 5 Division of Emergency Medicine, CNH, Washington, DC USA; 6 Deptartment of Biostatistics and Bioinformatics, Milken Institute School of Public Health, GWU, Washington, DC USA; 7 Deptartment of Pediatrics, University of Colorado Anschutz Medical Campus, Aurora, CO USA

**Keywords:** Paediatric research, Cystic fibrosis, Translational research

## Abstract

Persons with cystic fibrosis (PwCF) suffer from pulmonary exacerbations (PEx) related in part to lung infection. While higher microbial diversity is associated with higher lung function, the data on the impact of short-term antibiotics on changes in microbial diversity is conflicting. Further, *Prevotella* secretes beta-lactamases, which may influence recovery of lung function. We hypothesize that sub-therapeutic and broad spectrum antibiotic exposure leads to decreasing microbial diversity. Our secondary aim was to evaluate the concerted association of beta-lactam pharmacokinetics (PK), antibiotic spectrum, microbial diversity, and antibiotic resistance on lung function recovery using a pathway analysis. This was a retrospective observational study of persons with CF treated with IV antibiotics for PEx between 2016 and 2020 at Children’s National Hospital; respiratory samples and clinical information were collected at hospital admission for PEx (E), end of antibiotic treatment (T), and follow-up (F). Metagenomic sequencing was performed; PathoScope 2.0 and AmrPlusPlus were used for taxonomic assignment of sequences to bacteria and antibiotic resistance genes (ARGs). M/W Pharm was used for PK modeling. Comparison of categorical and continuous variables and pathway analysis were performed in STATA. Twenty-two PwCF experienced 43 PEx. The study cohort had a mean age of 14.6 years. Only 12/43 beta-lactam courses had therapeutic PK, and 18/43 were broad spectrum. A larger decrease in richness between E and T was seen in the therapeutic PK group (sufficient − 20.1 vs. insufficient − 1.59, *p* = 0.025) and those receiving broad spectrum antibiotics (broad − 14.5 vs. narrow − 2.8, *p* = 0.030). We did not detect differences in the increase in percent predicted forced expiratory volume in one second (ppFEV1) at end of treatment compared to PEx based on beta-lactam PK (sufficient 13.6% vs. insufficient 15.1%) or antibiotic spectrum (broad 11.5% vs. narrow 16.6%). While both therapeutic beta-lactam PK and broad-spectrum antibiotics decreased richness between PEx and the end of treatment, we did not detect longstanding changes in alpha diversity or an association with superior recovery of lung function compared with subtherapeutic PK and narrow spectrum antimicrobials.

## Introduction

There are more than 70,000 children and adults with living with cystic fibrosis (CF) worldwide^1^, and those with this progressive disease frequently suffer from recurrent episodes of lung infection and inflammation called pulmonary exacerbations (PEx)^2^. *Pseudomonas aeruginosa* and other Gram negative organisms are frequent opportunistic pathogens in this population^3^, and unfortunately beta-lactam antibiotic therapy directed against these pathogens is underdosed about 50% of the time^4–6^. Understanding the potential impact of insufficient beta-lactam antibiotic exposure on lung function is important to guide clinical use of beta-lactams^7–10^, especially in light of unclear evidence regarding the impact of antimicrobial resistance in persons with CF^11,12^.

While the relationship between *P. aeruginosa* or other Gram-negative pathogens and clinical outcomes is often evaluated as a single entity or in combination with a single pathogen^13–18^, it is important to consider that these pathogens exist within a microbial community^19,20^. Prior studies have repeatedly demonstrated an association between low microbial diversity and low lung function^21,22^. However, the contribution of antibiotics to changes in microbial diversity remains unclear. Long-term and large cross-sectional studies have demonstrated an association between increased antibiotic exposure and decreased microbial diversity^23,24^. Short-term studies of antibiotic treatment of PEx have shown short-term changes in diversity but this was not sustained at follow up^22,25^. Importantly, these prior studies did not include the variables of beta-lactam antibiotic exposure^26,27^, antibiotic spectrum^28,29^, nor the impact of anaerobes^30^ or community level antibiotic resistance^31,32^, such as the ability of *Prevotella* species to secrete beta-lactamases^33,34^.

Our overarching study hypothesis is that insufficient beta-lactam pharmacokinetics/pharmacodynamics (PK/PD; hereafter referred to as PK) and broad-spectrum antibiotic exposure leads to decreasing microbial diversity over time by fostering an environment for select bacteria to dominate the microbiome. Our secondary study aim was to evaluate the concerted association of beta-lactam PK and antibiotic spectrum, through impacts on microbial diversity (including *Prevotella* relative abundance) and beta-lactam antibiotic resistance, on recovery of pulmonary function.

## Materials and methods

### Study design

This was a prospective observational study of persons with CF treated with intravenous (IV) antibiotics for PEx between 2016 and 2020 at Children’s National Hospital in Washington DC (see Supplemental Methods). IRB approval was obtained from Children’s National Hospital (Pro6781, 8 Dec 2015 and Pro10528, 31 Aug 2018). Participants ≥ 18 years old provided written informed consent, and written parental informed consent was obtained for participants < 18 years old. Assent was obtained from children between the ages of 11–17 years. All research was performed in accordance with the Declaration of Helsinki. Respiratory samples and clinical information were collected from the data- and bio-repository on dates that coincided with hospital admission for PEx (E), end of antibiotic treatment (T), and their next follow-up visit (F), given that no additional antibiotics were administered between the T and F time points.

### Pharmacokinetics (PK) modeling

M/W Pharm (Version 3.80, Mediware) was used for PK modeling^35^. PK modeling of beta-lactam antibiotics was performed as previously published^6,26,27^, but briefly a beta-lactam population PK model was used that incorporated study participant age, weight, height, and serum creatinine at PEx, antibiotic dose, and antibiotic dosing schedule (see Supplemental Methods). The PK model was generated using the metadata above and did not incorporate directly measured serum samples.

### Respiratory sample collection and processing

Sputum, oropharyngeal (OP) swabs, and BAL specimens were collected and processed according to standard procedures as previously described (see Supplemental Methods)^26,27,36^.

### Bacterial DNA extraction, metagenomic sequencing, and bacterial load PCR

Bacterial DNA extraction, sequencing and PCR were performed as previously described (see Supplemental Methods)^21,27,37^. Between 23 and 30 libraries per run were sequenced on a NextSeq 500 (Illumina) using a Mid-Output 2 × 150 cycle kit. For analysis of the bacterial load, the run was considered successful if the cycle threshold (CT) of the sample run in triplicate was within one cycle. The mean bacterial load of the sample in triplicate was used for subsequent analyses. A log reduction between time points was used to analyze the change in bacterial load between samples.

### Bioinformatic analyses

FastQC and Flexbar were used to evaluate sequences for quality and for sequence trimming prior to putting sequences into downstream applications^38^. KneadData was used to filter out human sequences^39^. PathoScope 2.0 was used for taxonomic assignment of sequences to bacteria^40^. AmrPlusPlus, a Galaxy pipeline, was used for taxonomic assignment of sequences to antibiotic resistance genes (ARGs)^41^. The taxonomic and antibiotic resistance gene count tables were filtered to bacterial species and antibiotic class, respectively, and were imported into Rstudio v3.6.1 for subsequent analyses. The Rstudio packages used for analysis included *DESeq2* v.1.24.0^42^, *ggplot2* v3.2.0^43^, *phyloseq* v.1.28.0^44^, and *vegan* v.2.5-6^45^. Permutational analysis of variance (PERMANOVA) was also performed in Rstudio using the *adonis* function for analysis and portioning sums of squares using Bray–Curtis dissimilarities. Repeated patient samples were controlled for using the *strata* function. For comparative analyses taken into STATA, the Morisita–Horn index, observed species, Shannon index, and the inverse Simpson’s index were calculated using Explicet v2.10.5^46^.

### Statistical analyses

Comparison of categorical and continuous variables were performed in STATA/IC (v15.1); richness and alpha diversity between groups were compared using random-effects GLS regression (*xtreg*) and controlling for repeat participant samples (*xtset*) using robust standard errors. Structural equation modeling for pathway analysis was also performed in STATA/IC (see Supplemental Methods and Supplemental Fig. 1).

## Results

### Study participants and baseline clinical parameters

Twenty-two study participants met study inclusion criteria during the study period (Table 1). The median age of study participants at their first PEx was 16.5 years (range 7–23 years).

**Table 1:** Demographics and clinical characteristics of study cohort.

Study participant characteristics	N = 22
Female sex (n, %)	9 (41%)
**Race (n, %)**	
Black	1 (5%)
White	13 (59%)
Other	8 (36%)
**Ethnicity (n, %)**	
Hispanic	8 (36%)
**CFTR genotype (n, %)**	
F508del homozygous	10 (45%)
F508del heterozygous	5 (23%)
Other	7 (32%)
**Comorbidities (n, %)**	
Reflux	21 (95%)
Pancreatic insufficiency	19 (86%)
Asthma/reactive airway disease	16 (73%)
Sinusitis	6 (27%)
CF related diabetes	4 (18%)
Age at first PEx (mean, SD)	14.6 (5.0)
BMI at first PEx (mean, SD)	19.5 (3.4)
BMI Z score at first PEx (mean, SD) (n = 19)	**− **0.06 (1.02)
BMI percentile at first PEx (mean, SD) (n = 19)	49.4 (29.9)
Best ppFEV1 6 months prior to first PEx (SD)	81.2 (27.0)
Best ppFVC 6 months prior to first PEx (SD)	92.0 (27.1)
Best ppFEF25-75 6 months prior to first PEx (SD)	64.7 (37.0)
**Disease stage at first PEx, based on best ppFEV1 6 months prior to first PEx (n, %)**	
Early (ppFEV1 > 70%)	14 (64%)
Intermediate (ppFEV1 40–70%)	7 (32%)
Advanced (ppFEV1 < 40%)	1 (4%)
# PEx contributing to study (mean, SD)	2.0 (1.0)
**Current culture results (n, %)**	
MSSA	6 (14%)
MRSA	8 (19%)
*Pseudomonas aeruginosa*	20 (47%)
*Achromobacter xylosoxidans*	1 (2%)
*Burkholderia cepacia* complex*	4 (9%)
*Burkholderia gladioli*	3 (7%)
GP organism, other^†^	1 (2%)
GN organism, other^‡^	3 (7%)
PK sufficient (n, %)	12 (28%)
Broad antibiotic spectrum (n, %)	18 (42%)
**Primary beta-lactam used (n, %)**^**‖**^	
Ceftazidime	16 (37%)
Cefepime	9 (21%)
Piperacillin-tazobactam	7 (16%)
Meropenem	11 (26%)

*B. cepacia complex = 3 *B. cepacia*, 1 *B. multivorans.*

^†^GP, other = *Streptococcus pyogenes.*

^‡^GN, other = *Acinetobacter baumanii* (n = 1), *Pseudomonas putida* (n = 1), Unidentified GNR (n = 1).

^‖^In two instances, meropenem + ceftazidime was administered for *Burkholderia* sp. Considered meropenem as the primary beta-lactam as the organism was resistant to ceftazidime but susceptible to meropenem or because was resistant to both and meropenem had lower MIC. In one instance, meropenem followed ceftazidime for *P. aeruginosa* (switched due to rash on day 9). Organism was resistant to ceftazidime but susceptible to meropenem, so used meropenem as primary for analysis. In one instance, meropenem + piperacillin/tazobactam was administered for *Burkholderia* sp. Considered meropenem as the primary beta-lactam as the organism was resistant to pip/tazo but susceptible to meropenem.

### Pulmonary exacerbation characteristics

The 22 study participants experienced a total of 43 PEx that were included in the analysis (Table 1). Data on two additional PEx were collected but excluded; in one case only 48 h of a beta-lactam was received, and in the other case the beta-lactam received was not an anti-pseudomonal agent (e.g., ceftaroline). The most frequent signs and symptoms at the time of PEx that occurred in the majority of cases were increased cough (n = 43, 100%), change in sputum (n = 32, 74%), decreased ppFEV1 and ppFEF25/75 by > 10% (n = 27, 63%), increased dyspnea (n = 25, 58%), and CXR changes concerning for infection (n = 22, 51%). The mean (SD) ppFEV1 at PEx was 64% (24.5%) and the mean (SD) ppFEF25-75 was 46.4% (29.0%). The anti-pseudomonal beta-lactam antibiotics used in the study were ceftazidime, cefepime, piperacillin/tazobactam, and meropenem (Table 1). Additional antibiotics administered for treatment of PEx include IV tobramycin (n = 27, 63%), IV vancomycin (n = 6, 14%), IV/PO trimethoprim/sulfamethoxazole (n = 5, 12%), IV aztreonam (n = 5, 12%), IV/PO levofloxacin (n = 3, 7%), IV/PO linezolid (n = 3, 7%), IV/PO ciprofloxacin (n = 2, 5%), IV/PO doxycycline (n = 2, 5%), IV amikacin (n = 2, 5%), and IV clindamycin (n = 1, 2%). The mean (SD) duration of antibiotic treatment was 15 (5.0) days. Steroids were also concurrently administered in 46% (n = 19) of the treatment courses. Baseline medications prior to PEx and viral co-infection are shown in Supplemental Table 1.

### PK and antibiotic spectrum determinations

A total of 47 beta-lactam agents were administered for the 43 PEx events (Table 1, Supplemental Results). Antibiotic spectrum was assessed as broad or narrow based on the broadest spectrum beta-lactam antibiotic received^28^. Eighteen courses were considered broad spectrum (e.g., received meropenem and/or piperacillin/tazobactam) while 25 courses were considered narrow spectrum (e.g., received ceftazidime or cefepime).

### Respiratory sample, microbiome, and bacterial load characteristics

The distribution of respiratory samples across time points is shown in Supplemental Fig. 2. When performing the bacterial load PCR, six samples were run multiple times but did not meet the criteria for the CT being within one cycle. In those cases, the outlier was removed from the last run (the outlier was chosen based on the results of the prior runs), and the mean was reported using the two remaining values. A total of 109 bacterial species were identified across all samples, with each sample having a range of 11–81 species identified. The most abundant bacterial species identified across all samples was *S. aureus* (mean relative abundance 20.1%), followed by *Rothia mucilaginosa* (12.5%) and *P. aeruginosa* (8.5%). Sample specific bacterial relative abundance, richness and alpha diversity are shown in Supplemental Figs. 3 and 4 and Supplemental Table 2. The differences between sample specific richness, alpha diversity, and based on beta-lactam PK and antibiotic spectrum are shown in Supplemental Tables 3 and 4, respectively. As 26% of study participants were receiving azithromycin at least 3 times weekly at baseline, we tested for its potential impact on richness and alpha diversity at PEx and found no association (species observed *p* = 0.848, Shannon *p* = 0.960, inverse Simpson *p* = 0.533). Similarly, we checked for an association with the most commonly received inhaled antibiotics, tobramycin (*n* = 11, 26%) and aztreonam (*n* = 5, 12%). We did not find an association with inhaled antibiotic use and richness or alpha diversity at PEx (species observed *p* = 0.681 and 0.913, Shannon *p* = 0.939 and 0.910, and inverse Simpson *p* = 0.923 and 0.856, respectively). We also evaluated the differential abundance of bacterial species between time points based on sequencing data. We found that the traditional CF pathogens *P. aeruginosa*, *S. aureus*, and *H. influenzae* significantly decreased after antibiotic treatment (Supplemental Fig. 5A). Similarly, two *Burkholderia* species (*B. pseudomallei* and *B. ubonensis*) were significantly decreased at follow up compared to PEx onset (Supplemental Fig. 5B).

### Beta-lactam PK and antibiotic spectrum associations with changes in alpha and beta diversity

**Figure 1:**
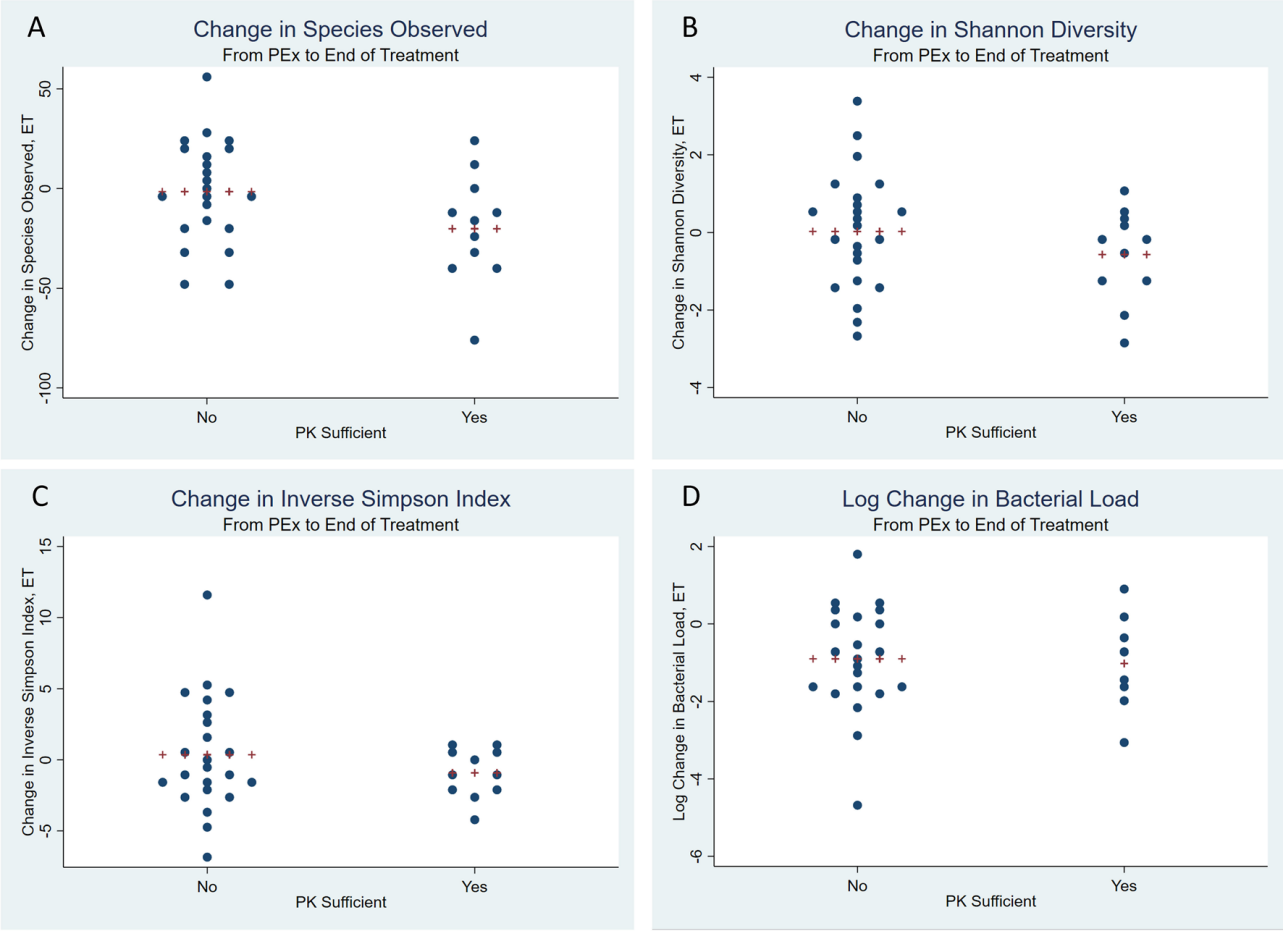
Change in alpha diversity and bacterial load by beta-lactam pharmacokinetic exposure from pulmonary exacerbation to end of antibiotic treatment. Panel (**A**) Change in Species Observed. Panel (**B**) Change in Shannon Diversity. Panel (**C**) Change in Inverse Simpson Index. Panel (**D**) Log Change in Bacterial Load. PK, pharmacokinetics; PEx, pulmonary exacerbation; E, pulmonary exacerbation; T, end of treatment; ET, change between pulmonary exacerbation and end of treatment. Plus signs represent the mean.

**Figure 2:**
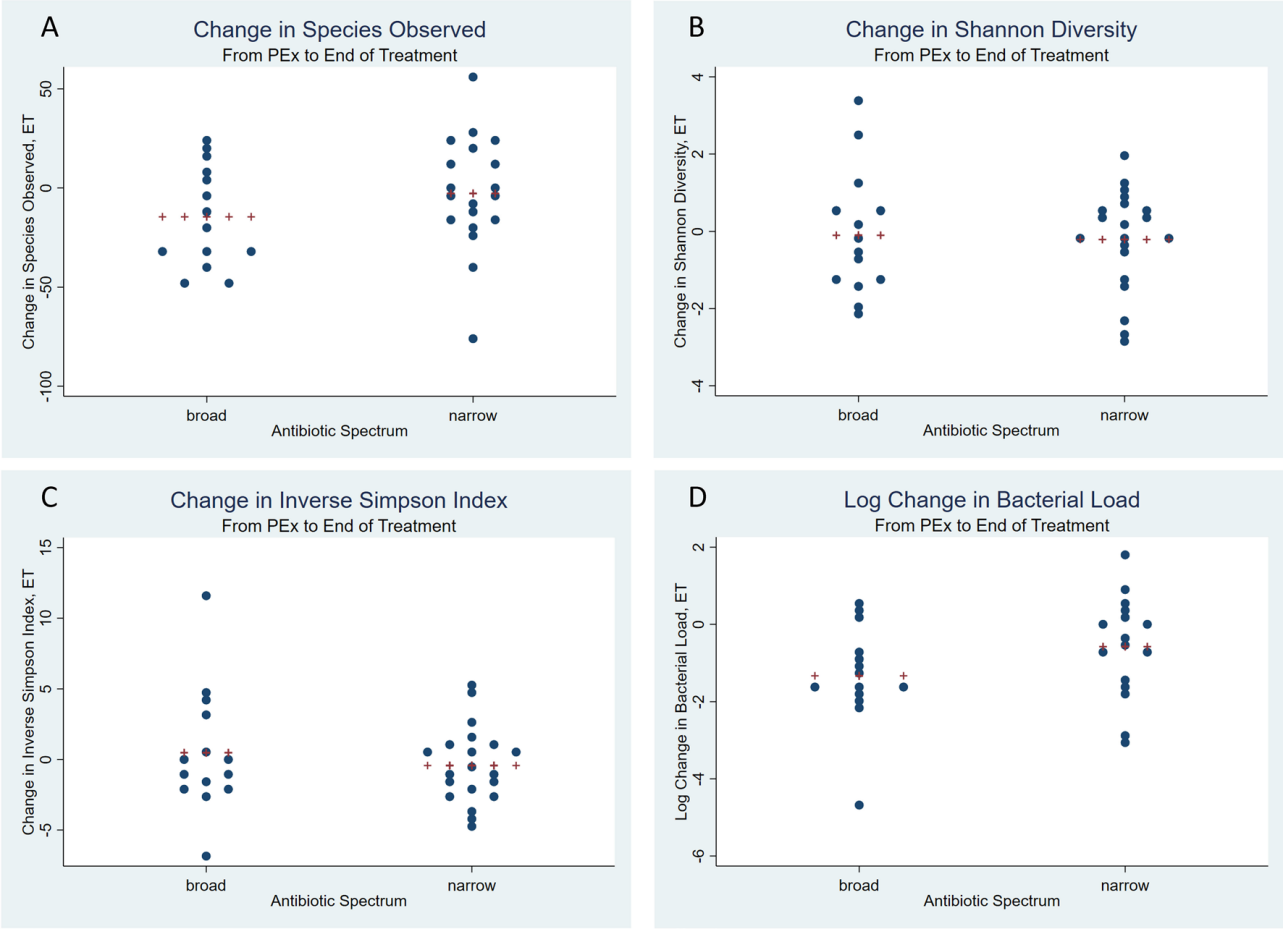
Change in alpha diversity and bacterial load by antibiotic spectrum exposure from pulmonary exacerbation to end of antibiotic treatment. Panel (**A**) Change in Species Observed. Panel (**B**) Change in Shannon Diversity. Panel (**C**) Change in Inverse Simpson Index. Panel (**D**) Log Change in Bacterial Load. PEx, pulmonary exacerbation; E, pulmonary exacerbation; T, end of treatment; ET, change between pulmonary exacerbation and end of treatment. Plus signs represent the mean.

The mean (SD) time between respiratory sample collection for the PEx (E) and end of treatment (T) sample was 11.8 (5.01) days. There were no differences noted based on beta-lactam PK (PK sufficient 11.3 vs. PK insufficient 12.0, *p* = 0.697) or antibiotic spectrum (broad 11.5 vs. narrow 12.1, *p* = 0.717). Likewise, there were no differences in the duration of antibiotic therapy based on beta-lactam PK (PK sufficient 14 vs. PK insufficient 15.9, *p* = 0.226) or antibiotic spectrum (broad 16.5 vs. narrow 14.5, *p* = 0.957). When comparing the change in the number of species observed between end of antibiotic treatment (T) and PEx onset (E) between beta-lactam PK sufficient and insufficient, we found a greater reduction in the richness of the PK sufficient group (a reduction of 20.1 species observed vs. a reduction of 1.6 species observed, *p* = 0.016; Fig. 1). No differences were observed in the change in Shannon diversity, the inverse Simpson index, or the log change in bacterial load. When comparing the change in the number of species observed between end of antibiotic treatment (T) and PEx onset (E) based on antibiotic spectrum, broad spectrum therapy had a greater reduction in species observed compared to narrow spectrum therapy, but this was not significant (a reduction of 14.5 species vs. a reduction of 2.8 species, *p* = 0.309; Fig. 2). No differences were noted in Shannon diversity, the inverse Simpson index, or the log change in bacterial load.

While there were no differences appreciated in the time between sample collection based on beta-lactam PK or antibiotic spectrum in our study cohort, it has been shown in other studies that the timing of sample collection during antibiotic therapy can affect outcomes^21^. Likewise, there was some overlap between the beta-lactam PK groups and the antibiotic spectrum groups that could affect our interpretation of the results. In addition to controlling for repeated patient samples, we performed a multivariate analysis incorporating beta-lactam PK, antibiotic spectrum, and a dichotomous variable for time between sample collected (< 7 days vs. ≥ 7 days). In the multivariate model, both beta-lactam PK and antibiotic spectrum were significantly associated with the change in microbial richness observed (*p* = 0.025 and *p* = 0.030, respectively).

The mean (SD) time between respiratory sample collection between for the PEx (E) and follow-up (F) sample was 68.8 (35.1) days. There were no differences noted based on beta-lactam PK (PK sufficient 74.7 vs. PK insufficient 61.6, *p* = 0.446). The time between sample collection was longer in those receiving broad spectrum antibiotics compared to those receiving narrow spectrum antibiotics (broad 84.8 vs. narrow 57.9, *p* = 0.030). However, no differences were noted in the change in richness or alpha diversity or the reduction in bacterial load based on beta-lactam PK (Supplemental Fig. 6) or antibiotic spectrum (Supplemental Fig. 7).

**Figure 3:**
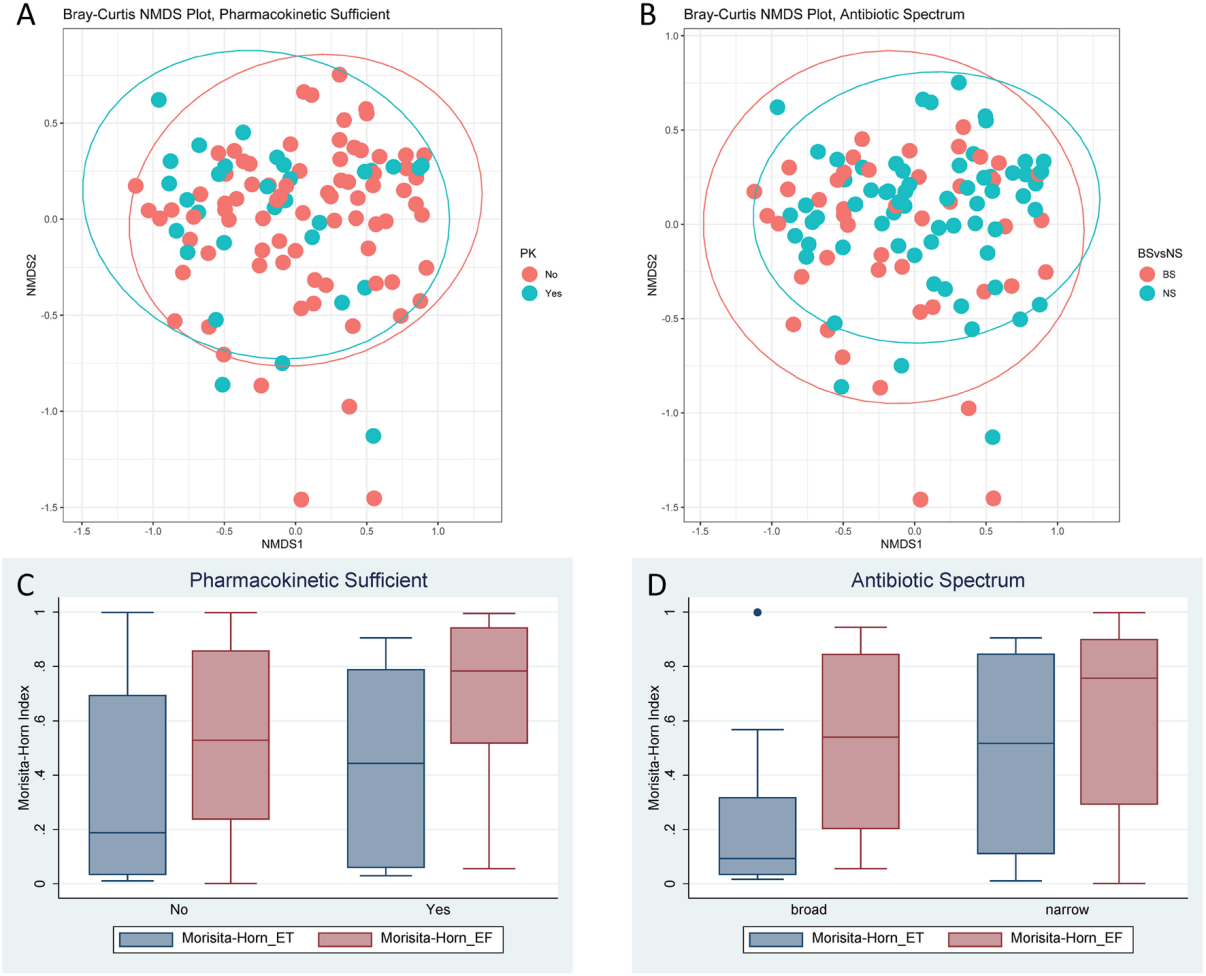
Changes in beta diversity. Panel (**A**) Bray–Curtis Non-metric multidimensional scaling (NMDS) Plot, Pharmacokinetic Sufficient. Panel (**B**) Bray–Curtis NMDS Plot, Antibiotic Spectrum. Panel (**C**) Morisita–Horn Index, Pharmacokinetic Sufficient. Panel (**D**) Morisita–Horn Index, Antibiotic Spectrum. NMDS, non-metric multidimensional scaling; PK, pharmacokinetic; E, pulmonary exacerbation; T, end of treatment; F, follow up; ET, change between pulmonary exacerbation and end of treatment; EF, change between pulmonary exacerbation and follow-up. On the NMDS plots, the ellipses represent the t distribution. The permutational multivariate analysis of variance (PERMANOVA) result for beta-lactam PK was *p* = 0.120 and for antibiotic spectrum was *p* = 0.133. On the box and whiskers plots, the center horizontal line corresponds to the median and the colored boxes represent the interquartile ranges. Small circles represent individual values that were outliers.

When looking at the change in microbial composition, we used both Bray–Curtis distances in a non-metric multidimensional scaling (NMDS) plot and the Morisita–Horn index (Fig. 3). The Bray–Curtis NMDS plots incorporated each sample and controlled for repeated patient samples. No differences were noted based on PK or antibiotic spectrum, with the caveat that these plots are likely more representative of inter-subject variability in the microbial composition. As the Morisita–Horn index was based on the difference between two samples, it was more representative of intra-subject changes. No differences were noted based on PK between either the PEx (E) and end of treatment (T) samples or the PEx (E) and follow up (F) samples. There was more dissimilarity seen in those receiving broad spectrum antibiotics compared to those receiving narrow spectrum antibiotics between the PEx (E) and end of treatment samples (T) (0.221 vs. 0.477, *p* = 0.036). This was not sustained in the comparison between PEx (E) and follow up (F) samples. When using the multivariate model as described for alpha diversity above, the impact of beta-lactam PK remained non-significant (*p* = 0.411), and the impact of antibiotic spectrum still approached significance (*p* = 0.060).

### Beta-lactam PK, antibiotic spectrum, and microbiome characteristics and associations with changes in pulmonary function

**Figure 4:**
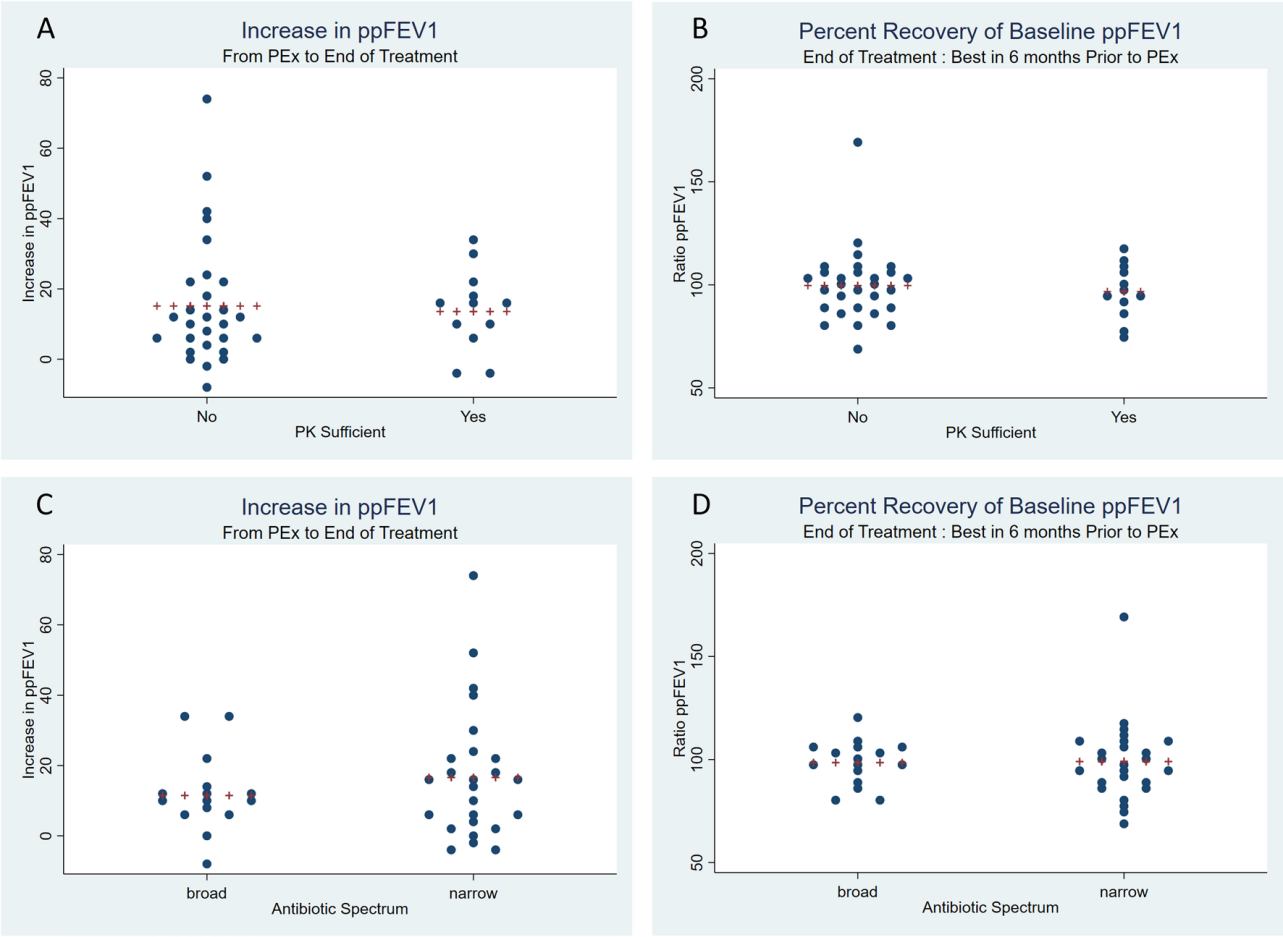
Recovery of pulmonary function. Panel (**A**) Increase in ppFEV1 from PEx to end of antibiotic treatment by beta-lactam pharmacokinetic exposure. Panel (**B**) Percent recovery of baseline ppFEV1 at end of treatment compared to the best ppFEV1 in the 6 months prior to PEx by beta-lactam pharmacokinetic exposure. Panel (**C**) Increase in ppFEV1 from PEx to end of antibiotic treatment by antibiotic spectrum exposure. Panel (**D**) Percent recovery of baseline ppFEV1 at end of treatment compared to the best ppFEV1 in the 6 months prior to PEx by antibiotic spectrum exposure. ppFEV1, percent predicted forced expiratory volume in one second; PEx, pulmonary exacerbation. Plus signs represent the mean.

Overall, the study participants had a significant improvement in their pulmonary function from PEx onset to end of treatment. Using a paired t-test, the mean ppFEV1 increased from 64.3 to 78.9% (*p* < 0.001), the mean ppFVC increased from 77.6 to 90.2% (*p* < 0.001), and the mean ppFEF25-75 increased from 46.7 to 61.9 (*p* < 0.001). Additionally, there were no significant differences in the end of treatment measures compared to the best in the prior 6 months (ppFEV1 79.1 vs. 80.1, *p* = 0.611; ppFVC 90.1 vs. 91.0, *p* = 0.584; ppFEF25-75 62.9 vs. 64.6, *p* = 0.525). For our pathway analysis, recovery of pulmonary function was measured as the increase in ppFEV1 from PEx onset (E) to end of treatment (T) and the ratio of the ppFEV1 at end of treatment (T) to the best ppFEV1 in the 6 months prior to PEx onset. Six separate structural equation models were run to incorporate the three different alpha diversity measures (species observed, Shannon index, and inverse Simpson index) as well as the two outcome measures. All models underwent several goodness of fit tests and all indices suggested good fitting models. Specifically, no model had a significant chi-square goodness of fit test, all RMSEA values were < 0.05, no pclose values were significant, and all comparative fit index values and Tucker–Lewis index values were > 0.95. The Z-score and *p* value for all of the model components are shown in Table 2. We did not detect a difference in the increase in ppFEV1 at end of treatment compared to PEx based on beta-lactam PK (sufficient 13.6% vs. insufficient 15.1%) or antibiotic spectrum (broad 11.5% vs. narrow 16.6%) (Fig. 4). We also did not detect a difference for percent recovery of ppFEV1 at end of treatment compared to the best ppFEV1 in the 6 months prior to the PEx (sufficient 96.8% vs. insufficient 99.6%; broad 98.3% vs. narrow 99.0%) (Fig. 4). Beta-lactam PK sufficient (yes/no) was inversely associated with species observed (*p* = 0.028), which was consistent with our baseline analyses using linear regression (Supplemental Table 1). There was an inverse relationship between the presence of beta-lactam antibiotic resistance genes and both Shannon diversity (*p* = 0.008) and the inverse Simpson index (*p* = 0.015). Lastly, there was a trend toward an inverse relationship between recovery of pulmonary function and the presence of antibiotic resistance genes in both recovery of baseline ppFEV1 (*p* = 0.083 for the Shannon diversity model and *p* = 0.068 for the inverse Simpson index model) and increase in ppFEV1 (*p* = 0.088 for the Shannon diversity model and *p* = 0.080 for the inverse Simpson index model).

**Table 2:** Structural equation modeling results.

	Model 1: Sobs, Ratio FEV1		Model 2: Sobs, Inc FEV1		Model 3: Shannon, Ratio FEV1		Model 4: Shannon, Inc FEV1		Model 5: SimpsonR, Ratio FEV1		Model 6: SimpsonR, Inc FEV1	
	Z score	*p* value	Z score	*p* value	Z score	*p* value	Z score	*p* value	Z score	*p* value	Z score	*p* value
**Diversity**												
PK	**2.20**	**0.028**	**2.20**	**0.028**	0.41	0.682	0.41	0.682	**− **0.70	0.482	**− **0.70	0.482
BS/NS	**− **1.49	0.136	**− **1.49	0.136	**− **0.08	0.939	**− **0.08	0.939	0.11	0.910	0.11	0.910
**Prevotella**												
PK	**− **1.11	0.268	**− **1.11	0.268	**− **1.11	0.268	**− **1.11	0.268	**− **1.11	0.268	**− **1.11	0.268
BS/NS	1.33	0.184	1.33	0.184	1.33	0.184	1.33	0.184	1.33	0.184	1.33	0.184
**BL ARG**												
PK	**− **0.69	0.488	**− **0.69	0.488	**− **0.69	0.488	**− **0.69	0.488	**− **0.69	0.488	**− **0.69	0.488
BS/NS	0.92	0.356	0.92	0.356	0.92	0.356	0.92	0.356	0.92	0.356	0.92	0.356
**PFTs**												
Diversity	1.24	0.216	**− **0.41	0.683	**− **0.70	0.486	**− **1.25	0.211	**− **0.88	0.378	**− **1.39	0.165
Prevotella	0.14	0.887	0.90	0.366	0.44	0.663	1.32	0.188	0.50	0.620	1.36	0.175
BL ARG	**− **1.44	0.150	**− **1.18	0.237	− 1.74	0.083	− 1.71	0.088	− 1.83	0.068	− 1.75	0.080
PK	**− **1.07	0.284	**− **0.24	0.811	**− **0.65	0.513	**− **0.31	0.753	**− **0.81	0.417	**− **0.58	0.560
BS/NS	0.63	0.527	1.10	0.272	0.40	0.692	1.32	0.188	0.41	0.682	1.33	0.183
**Cov**												
Div/Prev	0.23	0.822	0.23	0.822	1.07	0.283	1.07	0.283	1.12	0.265	1.12	0.265
Div/ARG	**− **0.58	0.564	**− **0.58	0.564	**− 2.67**	**0.008**	**− 2.67**	**0.008**	− **2.42**	**0.015**	− **2.42**	**0.015**
Prev/ARG	1.61	0.107	1.61	0.107	1.61	0.107	1.61	0.107	1.61	0.107	1.61	0.107

*BL* Beta-lactam; *ARG* Antibiotic resistance genes; *PFTs* Pulmonary function tests; *Cov* Covariance; *PK* Pharmacokinetics; *BS*/*NS* Broad spectrum versus narrow spectrum; *Prev Prevotella* sp.; *Sobs* Species observed; *SimpsonR* Inverse Simpson index; *Ratio FEV1* Ratio of the percent predicted forced expiratory volume in one second (ppFEV1) at end of treatment compared to the best ppFEV1 in the prior 6 months; Inc FEV1, increase in ppFEV1 at the end of treatment compared to pulmonary exacerbation onset.

Significant values are in [bold].

### Role of non-beta-lactam antibiotics on microbial diversity

We also examined the role of other antibiotics received during PEx treatment on the change in richness and alpha diversity measures from PEx to end of treatment and from PEx to follow up (Supplemental Table 5). Tobramycin was the most frequently used antibiotic (n = 27 of 43 courses) and was found to be significantly associated with the change in Shannon Diversity between PEx and end of treatment (tobramycin yes − 0.523 vs. no 0.406, *p* = 0.026). Vancomycin was the next most frequently used antibiotic (n = 6 of 43 courses) and was found to be significantly associated with both the change in species observed and inverse Simpson index between PEx and follow-up (vancomycin yes − 13.3 vs. no 10. 1, *p* = 0.003, and yes − 0.233 vs. no 1.317, *p* = 0.021, respectively). Aztreonam was used in 5 courses and had a significant impact on the change in Shannon diversity from PEx to end of treatment (aztreonam yes 0.749 vs. no − 0.0286, *p* < 0.001). Lastly, trimethoprim/sulfamethoxazole use in 5 courses was associated with a significant change in species observed from PEx to follow-up (trim/sulfa yes 22.3 vs. no 6.2, *p* = 0.004).

As each PEx was treated with multiple antibiotics, we attempted a multivariate analysis to explore if any specific antibiotics remained as significant variables associated with changes in richness and alpha diversity (Supplemental Table 6). Given the small numbers for some of the antibiotic courses, any significant findings need to be interpreted with caution. However, we did find that vancomycin continued to show significance for the change in inverse Simpson index between PEx and follow up (*p* = 0.001), whereas the other changes noted above did not remain significant. In the multivariate analysis, it was interesting that most of the significant variables were typically agents that would be used in MRSA infection (vancomycin, doxycycline, linezolid, and trimethoprim/sulfamethoxazole).

## Discussion

In our study of persons with CF receiving IV beta-lactam antibiotics for PEx with lower respiratory samples obtained at PEx onset, we found that both insufficient beta-lactam PK and broad-spectrum antibiotics were associated with a greater decrease in species richness at the end of antibiotic therapy compared to PEx onset. The microbial community also changed more in individuals receiving broad-spectrum antibiotics (e.g., a greater change intra-person beta-diversity) between PEx onset and the end of antibiotic therapy compared to those receiving narrow-spectrum antibiotics. While we found short-term changes, we did not find sustained changes when we compared PEx onset to follow-up after PEx resolution, suggesting the impacts in this study cohort were transient. We also did not find an association between beta-lactam PK or antibiotic spectrum and recovery of lung function in a model that incorporated microbial diversity, the relative abundance of *Prevotella* species, and the relative abundance of beta-lactam antibiotic resistance genes. Interestingly, in the analysis of relationships between variables, an increased presence of beta-lactam antibiotic resistance genes was associated with lower microbial diversity and lower lung function.

In prior studies evaluating the association between beta-lactam PK, our lab found that insufficient beta-lactam PK was associated with reduced short-term decreases in microbial diversity compared to sufficient beta-lactam dosing^26^. This is in direct contrast to the findings of this study, where insufficient beta-lactam PK was associated with a greater short-term decrease in microbial diversity. However, the prior study was limited by study participants in the insufficient PK group starting out with lower diversity and having more advanced lung disease; thus, the conclusion was they had less ability to decrease their diversity further. Additionally, this study incorporated NGS as opposed to 16S sequencing, which allowed for better refinement of the microbial community to the species level and increased the effect size our study had the power to detect. Another study of persons with CF using NGS found an association between sufficient beta-lactam PK and reduced microbial diversity similar our current findings, although the prior study found the change to be demonstrated in the comparison between the PEx onset and follow-up time points^27^. Together, these studies support the notion that the cumulation of individual antibiotic courses likely affect microbial diversity, but that the effect size is small enough that it would require a very large study to be powered sufficiently to detect these changes on a course-by-course basis. The association of antibiotic spectrum on intra-person beta-diversity found in this study confirms prior work in our lab^28^. This is not surprising, as broad-spectrum antibiotics by their definition have activity against Gram-negative, Gram-positive, and anaerobic organisms in the airway and thus are more likely to affect the microbial community at large.

We also noted a few interesting findings regarding bacterial species that were differentially abundant between time points. We found *Burkholderia pseudomallei* and *B. ubonensis* to be differentially abundance in PEx samples compared to end of treatment samples based on sequencing data. *B. pseudomallei* has previously been reported as a global pathogen in persons with CF, albeit more common in norther Australia or southeast Asia^47^. However, it should be noted that *B. pseudomallei* only contributed to 0.05% of all reads (85 K and of 185 million across all samples), whereas the other *Burkholderia* species (*B. cepacia*, *B. gladioli*, and *B. ubonensis*) were found to contribute more overall (2.4%, 0.88%, and 1.5%, respectively). Additionally, only *B. cepacia* and *B. gladioli* were identified in associated clinical cultures.

Studies to understand the nuances of antibiotic treatment on recovery of lung function have been limited by the complexity of the study population, the differing microbial pathogens that are targeted, and the variability in the antibiotics that are selected for treatment. Numerous articles have been published with contrasting results on the impact of antibiotic resistance on pulmonary outcomes^48^, as well as beta-lactam PK^6,10,28^ and antibiotic spectrum^28,29^. We sought to develop a model that wound incorporate these features, as well as microbial diversity since it has also been strongly associated with lung function^21,22,24^. Our model did not demonstrate an association with beta-lactam PK nor antibiotic spectrum with lung function recovery around the time of a single pulmonary exacerbation.

While not the primary or secondary outcomes of our study, we did identify associations between the relative abundance of antibiotic resistance genes detected by NGS and alpha diversity, as well as potential association between antibiotic resistance and recovery of pulmonary function. Because of the associations previously described between microbial diversity and antibiotic exposure^23,24^, and the knowledge that bacteria frequently adapt to antibiotic exposure by developing mechanisms of antibiotic resistance^49,50^, it would be reasonable to assume that this would be an expected outcome. The potential association between antibiotic resistance genes and pulmonary function is interesting, given the lack of association between antibiotic resistance detected via conventional methods and lung function recovery^48^ and the paradox this presents for clinical practice^12^. Our prior work and others^30–33^ have suggested the importance of incorporating community level resistance into an assessment of antibiotic resistance, and this study supports the need for more research in this area.

Lastly, we also examined the role of other antibiotics and the impact on changes in microbial diversity over time. Tobramycin was used in 63% of treatment courses, and its use was associated with a decrease in Shannon diversity at end of treatment. Changes were also noted with the use of vancomycin, aztreonam, and trimethoprim/sulfamethoxazole, but as they were much less frequently used (11–13% of courses), their true impact is less clear. In a multivariate analysis, vancomycin remained a significant variable in the change in species observed between PEx and follow-up. Other agents that also were also significant in the multivariate model were primarily anti-MRSA therapies (doxycycline, linezolid, and trimethoprim/sulfamethoxazole). This suggests that the link between MRSA infection, antibiotic treatment, and microbial diversity should be an area of future study.

There are several limitations to note in the interpretation of our results. First, the study was performed based on PEx occurrence before the widespread use of highly effective CFTR modulators, and no study participants were on elexacaftor-tezacaftor-ivacaftor at the time of PEx. Given the known impacts of elexacaftor-tezacaftor-ivacaftor on pulmonary function^51,52^ and emerging data suggesting changes in the microbial ecology of the airway^53^, there may be differences in persons with CF on this treatment. Second, as this was a single center study and limited to children and young adults who could spontaneously produce sputum, the total number of study participants is small and skews toward those with more symptomatic disease, which may impact the generalizability of these results. However, almost two thirds (64%) of the study participants were considered early disease stage based on their ppFEV1 prior to their first PEx. Our a priori power analyses were based on the estimation that 50% of study participants would be beta-lactam PK insufficient, but in this study 72% of study participants met that criterium. This affects our power to detect changes in both microbial diversity and lung function and may have impacted our findings. We also attempted to further parse out the concerted versus individual impact of all antibiotics that were used for treatment of PEx. This again was limited by the sample size of our study. Lastly, there are a few points about sequencing data that should be noted. We found many bacterial species within the sequencing data that were not present in clinical cultures. This is likely due to several factors, including the culture conditions within the clinical lab (i.e., not ideal for anaerobic growth) or due to misalignment of sequencing data to the representative clades. We should also note that while we had excellent sequencing depth to explore bacterial species and thus diversity, our assessment of antibiotic resistance genes is more limited.

In summary, both sufficient beta-lactam PK and broad antibiotic spectrum were associated with short-term reductions in bacterial species richness at the end of antibiotic therapy, but these differences were no longer apparent at follow up. Similarly, changes in beta diversity as measured by the Morisita–Horn index were increased by broad antibiotic spectrum, but those differences were not apparent at follow up. In a model incorporating microbial diversity, the relative abundance of *Prevotella* species, and the presence of beta-lactam antibiotic resistance genes, neither beta-lactam PK nor antibiotic spectrum were associated with differences in the recovery of pulmonary function. The impact of community level antibiotic resistance on clinical outcomes should be explored in future studies.

## Supplementary Information

Supplementary Information 1.

Supplementary Information 2.

## Data Availability

The sequence dataset supporting the conclusions of this article is available in the NCBI SRA repository under BioProject PRJNA825831. The batch and R scripts used for bioinformatic analyses have been uploaded to GitHub (github.com/alhahn/CF_Shwachman).
