# Supplementary material for: Therapeutic beta-lactam dosages and broad-spectrum antibiotics are associated with reductions in microbial richness and diversity in persons with cystic fibrosis
Source: Sci Rep. 2023 Jan 21;13:1217. doi: 10.1038/s41598-023-27628-x (PMC9867719; doi:10.1038/s41598-023-27628-x)
Supplement: Supplementary file 1 — Supplementary Information 1. [file 41598_2023_27628_MOESM1_ESM.pdf]

Therapeutic beta-lactam dosages and broad-spectrum antibiotics are associated with reductions in microbial richness and diversity in persons with cystic fibrosis

Andrea Hahn, Aszia Burrell, Hollis Chaney, Iman Sami, Anastassios C. Koumbourlis, Robert J. Freishtat, Keith A. Crandall, and Edith T. Zemanick

Supplemental Methods

Study design. To be included in this study, the respiratory sample at PEx (E) had to be deep respiratory sample (e.g., sputum or bronchoalveolar lavage (BAL)). Clinical information collected included demographics (age, weight, height, race, ethnicity, and CFTR mutation type), underlying comorbidities, baseline medication use, current and prior respiratory culture results, laboratory values (including serum creatinine at time of PEx), and pulmonary function tests. The best percent predicted forced expiratory volume in one second (ppFEV1) in the 6 months prior to the PEx was used to define baseline lung function and the study participant's disease stage [1]. PEx was defined based on treatment with IV antibiotics and using modified Fuchs' criteria [2], with presentation with the following signs and symptoms noted: increased cough, increased sputum production, hemoptysis, increased sinus discharge, sinus pain, increased dyspnea, malaise or fatigue, anorexia or weight loss, fever > 38°C, change in exam of the chest, decrease in ppFEV1 ≥10% from the best ppFEV1 in the prior 6 months, or radiographic changes.

Pharmacokinetics (PK) modeling. For adults >20 years of age at the time of PEx (n=6), creatinine clearance was calculated using the Cockcroft & Gault equation [3,4]. For children and young adults ≤ 20 years of age at the time of PEx (n=37), the Schwartz equation was used [4,5]. The time above the minimum inhibitory concentration (T>MIC) at the 4th, 8th, and 12th doses were measured and averaged. The MIC used for measurement was based on the Gram-negative organism grown in the PEx culture. If the patient was receiving two beta-lactams, the one with the lowest MIC was used for determination of T>MIC. For those where a Gram-negative organism was not present in the PEx culture, all had a prior history of Gram-negative infection. As such, for those participants the MIC of the most recent Gram-negative organism was used for T>MIC determination. Lastly, to be considered beta-lactam PK sufficient, the T>MIC for cephalosporins had to be ≥60% of the dosing interval, for penicillins ≥50% of the dosing interval, and for carbapenems ≥40% of the dosing interval [6].

Respiratory sample collection and processing. Sputum and BAL specimens are collected in sterile containers and OP swabs are collecting using Eswab with Amies media (Copan). All specimens are stored at 4°C until sample processing. Sputum and BAL specimens are mixed 1:1 volume/volume with Sputasol (ThermoFisher) and sterile normal saline, vortexed, and heated in a 37°C heated bead bath for 15 minutes to homogenize the sample. Homogenized sputum and BAL samples and the Amies media from Eswabs are transferred to microcentrifuge tubes and pelleted through centrifugation (12,000g x 10 minutes). Supernatants are removed, and pellets and supernatants are stored separately at -80°C.

Bacterial DNA extraction and metagenomic sequencing. Frozen cell pellets were thawed and mixed with sterile phosphate buffered saline (PBS) prior to DNA extraction using a QIAamp DNA Microbiome Kit (Qiagen). This kit enzymatically depletes free DNA (i.e., human DNA in the mixed sample) using benzonase followed by proteinase K before physically (pathogen lysis tubes) and enzymatically (again

proteinase K) opening the bacterial cell walls/outer membranes to extract the bacterial DNA. Qubit (Thermofisher Scientific) and Bioanalyzer (Agilent) were used to measure DNA quantity and quality, respectively. DNA libraries for next generating sequencing (NGS) were constructed using a Nextera XT Library Prep Kit (Illumina). Between 23-30 libraries per run were sequenced on a NextSeq 500 (Illumina) using a Mid-Output 2x150 cycle kit. We had an average of 5.8 million reads/sample (range 670K to 21M). After filtering out the remaining human reads in the samples using KneadData [7], we were left with an average of 1.6 million reads/sample (range 38K to 8M). For each individual sample, our Goods coverage was 1, supporting that our sequencing depth was sufficient for subsequent analysis of microbial diversity measures.

Determination of beta-lactam antibiotic resistance genes. AMR Plus Plus [8] was used to identify beta-lactam resistance genes. This program uses the MEGARes database (https://megares.meglab.org/), which contains the sequence data for more than 8,000 antimicrobial resistance genes. Specifically for beta-lactams, this includes Class A-D beta-lactamases, mutant porin proteins, penicillin binding protein, and penicillin binding protein regulator. The resulting count table for beta-lactam resistance as an antibiotic class was used for subsequent statistical analyses.

Bacterial load PCR. Total 16S rRNA gene copy number was measured using quantitative real-time PCR to determine the bacterial load within each sample. Primers used include the forward primer 5'-TCCTACGGGAGGCAGCAGT-3', the reverse primer 5'-GGACTACCAGGGTATCTAATCCTGTT-3', and the probe (6-FAM)-5'-CGTATTACCGCGGCTGCTGGCAC-3' (TAMRA) [9,10].

Statistical analyses. The exogenous variables in the structural equation model were beta-lactam PK sufficient (yes/no) and antibiotic spectrum (broad versus narrow). The first endogenous variable in the model was alpha diversity of the respiratory sample obtained at PEx onset (E), and the model was run using species observed, the Shannon index, and the inverse Simpson index. The other two endogenous variables were the relative abundance of *Prevotella* sp. and the relative abundance of beta-lactam antibiotic resistance genes in the PEx sample (E). The outcome variables were the increase in the percent predicted forced expiratory volume in one second (ppFEV1) from PEx onset (E) to end of antibiotic treatment (T) and the percent recovery of ppFEV1 at the end of antibiotic treatment (T) compared to the best ppFEV1 in the 6 months prior to PEx onset. A maximum likelihood with missing values model was used, with default standard errors.

Supplemental Results

Beta-lactam PK determinations. In two instances, meropenem and ceftazidime were administered concurrently and in one instance, meropenem and piperacillin/tazobactam were administered concurrently, all for treatment of *Burkholderia* sp. In all situations, meropenem was considered as the primary beta-lactam as the organism was resistant to the other beta-lactams (n=2) or meropenem had lower MIC (n=1). In one instance, meropenem was discontinued after 9 days of treatment due to development of rash and the study participant was started on ceftazidime. As the organism was resistant to ceftazidime but susceptible to meropenem, meropenem was used as primary beta-lactam for analysis. Additionally, the antimicrobial susceptibility results from the Gram-negative organisms used to determine T>MIC were obtained from the concurrent PEx respiratory culture for 67% (n=29) of the PEx event. The other 33% (n=14) were based on a Gram-negative organism in a prior culture (*P. aeruginosa*, n=13 and *S. maltophilia,* n=1). Twelve beta-lactam treatment courses achieved a T>MIC associated with bacterial killing and thus considered PK sufficient, while the remaining 31 courses were considered PK insufficient. As *S. aureus* was also detected in 14 PEx cultures, of which 8 were MRSA, the combination of antibiotics administered was also assessed for therapy that also targeted *S. aureus*. The antibiotics selected did not cover for MRSA in one instance, but this course was already deemed PK insufficient and so no changes were required to our assigned groups.

Beta-lactam antibiotic resistance genes. More than 900 antibiotic resistance genes were identified in our sample cohort (please see the supplemental Excel spreadsheet). Of these, 292 (32% of the total genes detected) were directed against beta-lactams. 26,638 total sequence hits (1.9%) were for Class A beta-lactamases, which included BLA, BLAZ, CARB, CFX, CTX, GES, HERA, MAL, OXY, ROB, SCO, SFO, SHV, and TEM. Importantly, 6229 of those sequence hits were against CFX, with *CfxA* being the most notable beta-lactamase associated with *Prevotella* species [11–13]. 15,576 total hits (1.1%) were for penicillin binding proteins, which included MECA, MECC, MECI, PBPB1A, PBP1B, BPB2, PBPB2B, PBP2X, and PBP4B. Only 5576 (0.4%) total hits were for Class B (16), Class C (2982), or Class D (2577) beta-lactamases. 2402 hits (0.17%) were for mutant porin proteins. As the total number of sequence hits for antibiotic resistance genes was much lower than for bacterial species, we observed that the rarefaction curves for antibiotic class suggested good coverage for comparison across samples, whereas we did not have enough sequencing depth to look at the antibiotic genes specifically. As such, beta-lactam resistance as an antibiotic class was used for subsequent analyses.

Association between azithromycin or inhaled antibiotic use and culture results. Azithromycin and inhaled antibiotics including tobramycin and aztreonam are typically started as chronic therapies in persons with CF persistently infected with *P. aeruginosa*. Other studies have suggested these therapies can be associated with the growth of other pathogens. Using chi-square, we tested the association between these agents and the top 5 pathogens identified in clinical culture: MSSA, MRSA, *P. aeruginosa*, *B. gladioli*, and *B. cepacia* complex (Supplemental Table 7). In our study cohort, only *P. aeruginosa* was associated with the use of these chronic antibiotic therapies.

Supplemental Table 1. Additional Baseline and Pulmonary Exacerbation Clinical Characteristics

Baseline Characteristics Prior to PEx	N=43
**Azithromycin use (n, %)**	11 (26%)
**Inhaled antibiotics (n, %)**	
**Tobramycin**	11 (26%)
**Aztreonam**	5 (12%)
**Continuous use of alternating inhaled antibiotics (n, %)**	5 (12%)
**CFTR Modulator Use (n, %)**	
**Ivacaftor**	5 (12%)
**Ivacaftor/lumacaftor**	11 (26%)
**Tezacaftor/ivacaftor**	2 (5%)
**PEx Characteristics**	**N=43**
**Concurrent respiratory virus results (n, %)**	
**Not tested**	21 (49%)
**Human rhino/enterovirus**	4 (9%)
**Respiratory syncytial virus**	2 (5%)
**Non-SARS coronavirus**	2 (5%)
**Influenza A***	1 (2%)
**Influenza B**	1 (2%)
**Parainfluenza***	1 (2%)

*These two viruses were a co-infection in one study participant

PEx, pulmonary exacerbation; CFTR, cystic fibrosis transmembrane conductance regulator; SARS, severe acute respiratory syndrome.

Supplemental Table 2. Alpha Diversity Measures by Time Point

PtID	PEx#	E_SObs	E_Shan	E_SimpR	T_SObs	T_Shan	T_SimpR	F_SObs	F_Shan	F_SimpR
**SH01**	1	81	2.937	4.056	41	2.764	4.074	78	3.815	8.932
**SH01**	2	76	4.028	9.255	42	2.020	2.497	87	3.257	4.225
**SH01**	3	20	1.123	2.044	24	1.221	2.070			
**SH02**	1	75	4.080	10.176				66	3.872	8.684
**SH02**	2	19	2.667	5.129	75	3.128	4.541			
**SH03**	1	31	2.627	4.815				76	4.359	12.968
**SH03**	2	52	3.141	4.990	64	2.893	4.028	77	1.429	1.475
**SH03**	3	47	1.281	1.667	58	3.326	6.192	81	3.910	10.154
**SH03**	4	13	2.832	5.983				80	2.704	2.978
**SH05**	1	40	3.353	6.807	38	3.102	4.381	44	2.933	4.498
**SH05**	2	52	1.911	2.351	47	2.270	2.978	54	2.001	2.611
**SH06**	1	79	3.473	4.560	66	2.206	2.222	68	4.335	13.367
**SH07**	1	44	2.597	3.007	31	0.497	1.150	80	3.822	6.375
**SH07**	2	46	3.913	7.506	13	2.618	4.835	75	2.262	2.173
**SH08**	1	57	2.946	3.725	38	0.711	1.187	76	4.101	8.847
**SH08**	2	70	4.211	10.855	53	4.039	9.130			
**SH09**	1	49	0.963	1.270	48	2.069	2.222	78	2.608	3.253
**SH09**	2	29	2.531	3.985	53	2.880	4.435	34	3.102	6.313
**SH09**	3	66	1.274	1.358				65	0.437	1.088
**SH10**	2	63	3.357	6.029	15	2.592	4.538	49	1.671	1.798
**SH10**	3	11	1.804	2.279	26	2.266	2.850	38	3.819	9.719
**SH11**	1	78	0.552	1.139	31	2.972	4.344	61	2.177	2.495
**SH11**	2	27	2.679	2.985	22	1.458	1.759	38	4.063	11.224
**SH11**	3	46	3.265	6.431	65	2.756	5.272	40	3.486	6.657
**SH12**	1	37	2.966	5.142	19	2.388	4.086			
**SH12**	2	35	3.086	6.261	53	2.660	2.557	24	2.216	3.438
**SH12**	3	37	3.432	7.825				51	3.516	7.209
**SH12**	4	59	3.848	8.625				38	3.300	6.454
**SH13**	1	26	2.479	3.085	51	3.655	7.579	44	3.141	5.009
**SH14**	1	75	2.863	4.218				70	1.669	1.739
**SH15**	1	34	2.659	3.205	59	3.878	8.678	69	4.157	9.241
**SH15**	2	54	3.459	6.821				45	2.997	4.975
**SH16**	1	55	2.322	3.006	16	2.548	3.715	44	2.041	2.803
**SH33**	1	59	2.791	3.320	38	3.294	7.391			
**SH34**	1	37	1.178	1.393				47	2.181	2.349
**SH34**	2	32	1.265	1.409	22	2.177	2.902	24	0.989	1.372
**SH34**	3	36	2.288	2.375	32	2.915	4.897	57	3.068	4.619

**SH38**	2	25	2.139	2.970	54	0.710	1.187	19	0.306	1.074
**SH38**	3	43	0.188	1.038				15	1.426	1.793
**SH39**	1	62	0.664	1.158				82	3.256	3.354
**SH42**	1	65	0.921	1.250	74	4.288	12.978	43	2.741	3.614
**SH43**	1	42	3.079	5.292	8	1.733	3.045			
**SH44**	1	54	2.193	2.957	29	2.808	3.785	53	3.345	5.609

E, pulmonary exacerbation; T, end of treatment; F, follow-up; SObs, species observed; Shan, Shannon index; SimpR, inverse Simpson index.

Supplemental Table 3. Baseline Alpha Diversity, Bacterial Load by Time Point based on Beta-Lactam Pharmacokinetic Exposure

	PK sufficient (n=12)	PK insufficient (n=31)	P value*
E Sobs (mean, SD)	55.6 (16.6) (range 29-81)	44.2 (18.8) (range 11-78)	**0.028**
E Shannon (mean, SD)	2.60 (0.8) (range 0.96-3.91)	2.46 (1.1) (range 0.19-4.21)	0.651
E Inverse Simpson (mean, SD)	3.84 (1.7) (range 1.27-7.51)	4.44 (2.9) (range 1.0-10.9)	0.410
E Bacterial Load (mean SD)	4.3e6 (6.2e6)	1.7e7 (3.4e7)	**0.087**
T Sobs (mean, SD)	38 (19.4) (range 13-66)	42.0 (18.5) (range 8-75)	0.472
T Shannon (mean, SD)	2.37 (0.7) (range 0.5-2.9)	2.60 (1.0) (range 0.71-4.29)	0.400
T Inverse Simpson (mean, SD)	3.46 (1.18) (range 1.15-4.84)	4.68 (3.0) (range 1.18-12.98)	0.160
T Bacterial Load (mean, SD)	5.7e5 (8.1e5)	2.2e6 (8.3e6)	0.418
F Sobs (mean, SD)	65.6 (15.4) (range 34-80)	51.8 (20.5) (range 15-87)	0.160
F Shannon (mean, SD)	2.62 (1.2) (range 0.44-4.33)	2.91 (1.0) (range 0.31-4.36)	0.492
F Inverse Simpson (mean, SD)	4.83 (3.8) (range 1.09-13.37)	5.44 (3.4) (range 1.07-12.97)	0.663
F Bacterial Load (mean, SD)	1.8e6 (1.8e6)	1.5e7 (3.7e7)	**0.093**

PK, beta-lactam pharmacokinetics; E, pulmonary exacerbation; Sobs, species observed; T, end of antibiotic treatment; F, follow up.

Supplemental Table 4. Baseline Alpha Diversity, Bacterial Load by Time Point based on Antibiotic Spectrum

	Broad (n=18)	Narrow (n=25)	P value*
E Sobs (mean, SD)	50.7 (21.0) (range 11-81)	45.0 (17.0) (range 13-79)	0.260
E Shannon (mean, SD)	2.49 (1.2) (range 0.55-4.08)	2.50 (0.95) (range 0.19-4.2)	0.994
E Inverse Simpson (mean, SD)	4.29 (2.8) (range 1.14-10.18)	4.26 (2.48) (range 1.04-10.86)	0.970
E Bacterial Load (mean SD)	1.2e7 (2.9e7)	1.5e7 (3.2e7)	0.759
T Sobs (mean, SD)	34.4 (19.2) (range 8-74)	45.8 (17.0) (range 16-75)	0.152
T Shannon (mean, SD)	2.44 (1.0) (range 0.50-4.29)	2.59 (0.87) (range 0.71-4.04)	0.595
T Inverse Simpson (mean, SD)	4.60 (3.1) (range 1.15-12.98)	4.06 (2.18) (range 1.19-9.13)	0.377
T Bacterial Load (mean, SD)	2.9e5 (5.7e5)	3.0e6 (9.7e6)	0.258
F Sobs (mean, SD)	58.2 (18.1) (range 38-87)	54.4 (21.5) (range 15-81)	0.776
F Shannon (mean, SD)	3.10 (0.7) (range 1.67-4.06)	2.63 (1.23) (range 0.31-4.36)	**0.093**
F Inverse Simpson (mean, SD)	5.44 (3.0) (range 1.80-11.22)	5.13 (3.8) (range 1.07-13.37)	0.733
F Bacterial Load (mean, SD)	1.7e7 (4.6e7)	6.8e6 (1.4e7)	0.396

E, pulmonary exacerbation; Sobs, species observed; T, end of antibiotic treatment; F, follow up.

Supplemental Table 5. Univariate Analysis of Other Antibiotics on Changes in Alpha Diversity

Additional Antibiotics Used*	Species Observed, ET	Species Observed, EF	Shannon Diversity, ET	Shannon Diversity, EF	Inverse Simpson Index, ET	Inverse Simpson Index, EF
Tobramycin (n=27)	p=0.744	p=0.253	**p=0.026**	p=0.671	p=0.079	p=0.942
Vancomycin (n=6)	p=0.337	**p=0.003**	p=0.958	p=0.714	p=0.514	**p=0.021**
Aztreonam (n=5)	p=0.245	p=0.621	**p<0.001**	p=0.993	p=0.168	p=0.841
Trimethoprim/ sulfamethoxazole (n=5)	p=0.474	**p=0.004**	p=0.399	p=0.583	p=0.554	p=0.545

*The following antibiotics were used in <10% of antibiotic courses so were excluded from comparisons: amikacin (n=2), ciprofloxacin (n=2), clindamycin (n=1), doxycycline (n=3), levofloxacin (n=3), and linezolid (n=3).

Supplemental Table 6. Multivariate Analysis of Beta-lactam Pharmacokinetics, Antibiotic Spectrum, and Individual Antibiotics on Changes in Alpha Diversity

Variable*	Species Observed, ET	Species Observed, EF	Shannon Diversity, ET	Shannon Diversity, EF	Inverse Simpson Index, ET	Inverse Simpson Index, EF
Beta-lactam PK	P=0.092	P=0.398	P=0.678	P=0.630	P=0.370	P=0.962
Antibiotic Spectrum	P=0.420	P=0.387	P=0.016	P=0.421	P=0.105	P=0.861
Aztreonam IV (n=5)	P=0.414	P=0.915	P=0.782	P=0.898	P=0.048	P=0.743
Ceftazidime IV (n=20)	P=0.522	P=0.553	P=0.761	P=0.470	**P<0.001**	P=0.937
Cefepime IV (n=9)	P=0.033	P=0.340	P=0.642	P=0.028	P=0.055	P=0.919
Piperacillin/ tazobactam IV (n=8)	P=0.049	P=0.276	P=0.346	P=0.087	**P=0.001**	P=0.395
Meropenem IV (n=11)	P=0.606	Omitted	P=0.175	Omitted	**P<0.001**	Omitted
Tobramycin IV (n=27)	P=0.919	P=0.479	P=0.800	P=0.608	**P=0.002**	P=0.787
Amikacin IV (n=2)	P=0.205	P=0.419	**P<0.001**	P=0.975	**P<0.001**	P=0.897
Ciprofloxacin IV (n=1)	**P=0.001**[†]	P=0.568	P=0.029	P=0.136	P=0.156	P=0.526
Levofloxacin IV (n=2)	P=0.352	Omitted	P=0.466	Omitted	P=0.150	Omitted
Vancomycin IV (n=6)	**P=0.001**	**P=0.001**	P=0.209	P=0.022	P=0.137	P=0.003
Linezolid IV (n=2)	P=0.303	P=0.287	P=0.898	P=0.390	P=0.023	P=0.332
Clindamycin IV (n=1)	Omitted*	Omitted	Omitted	Omitted	Omitted	Omitted
Doxycycline IV (n=1)	**P<0.001**	**P<0.001**	**P<0.001**	**P<0.001**	**P<0.001**	**P<0.001**
Trimethoprim/ sulfamethoxazole IV (n=1)	P=0.247	Omitted	P=0.898	Omitted	**P=0.001**	Omitted
Ciprofloxacin PO (n=2)	P=0.010	P=0.031	P=0.344	P=0.246	P=0.094	P=0.988
Levofloxacin PO (n=1)	**P<0.001**	P=0.130	P=0.345	P=0.039	P=0.014	**P<0.001**
Linezolid PO (n=1)	P=0.879	**P<0.001**	P=0.179	**P<0.001**	P=0.174	**P<0.001**
Doxycycline PO (n=2)	Omitted	P=0.024	Omitted	P=0.814	Omitted	P=0.616
Trimethoprim/ sulfamethoxazole PO (n=5)	**P<0.001**	P=0.032	P=0.721	P=0.004	P=0.004	P<0.012

*Omitted by the GLS model because of collinearity

[†]Bolded p values are considered significant and are p values of 0.002 or less, intending to account for a false discovery rate given the use of 21 variables in this model

IV, intravenous; PO, by mouth

Supplemental Table 7. Association Between Chronic Antibiotics and PEx Culture Results

PEx Culture Results	Azithromycin (n=11)	Inhaled Tobramycin (n=11)	Inhaled Aztreonam (n=5)
P. aeruginosa (n=20)	9 with both; **p=0.006**	9 with both; **p=0.006**	5 with both; **p=0.011**
MRSA (n=8)	1 with both; p=0.347	1 with both; p=0.347	0 with both; p=0.255
MSSA (n=6)	0 with both; p=0.122	0 with both; p=0.122	0 with both; p=0.338
B. cepacia complex (n=4)	2 with both; p=0.267	0 with both; p=0.218	0 with both; p=0.446
B. gladioli (n=3)	0 with both; p=0.292	0 with both; p=0.292	0 with both; p=0.515

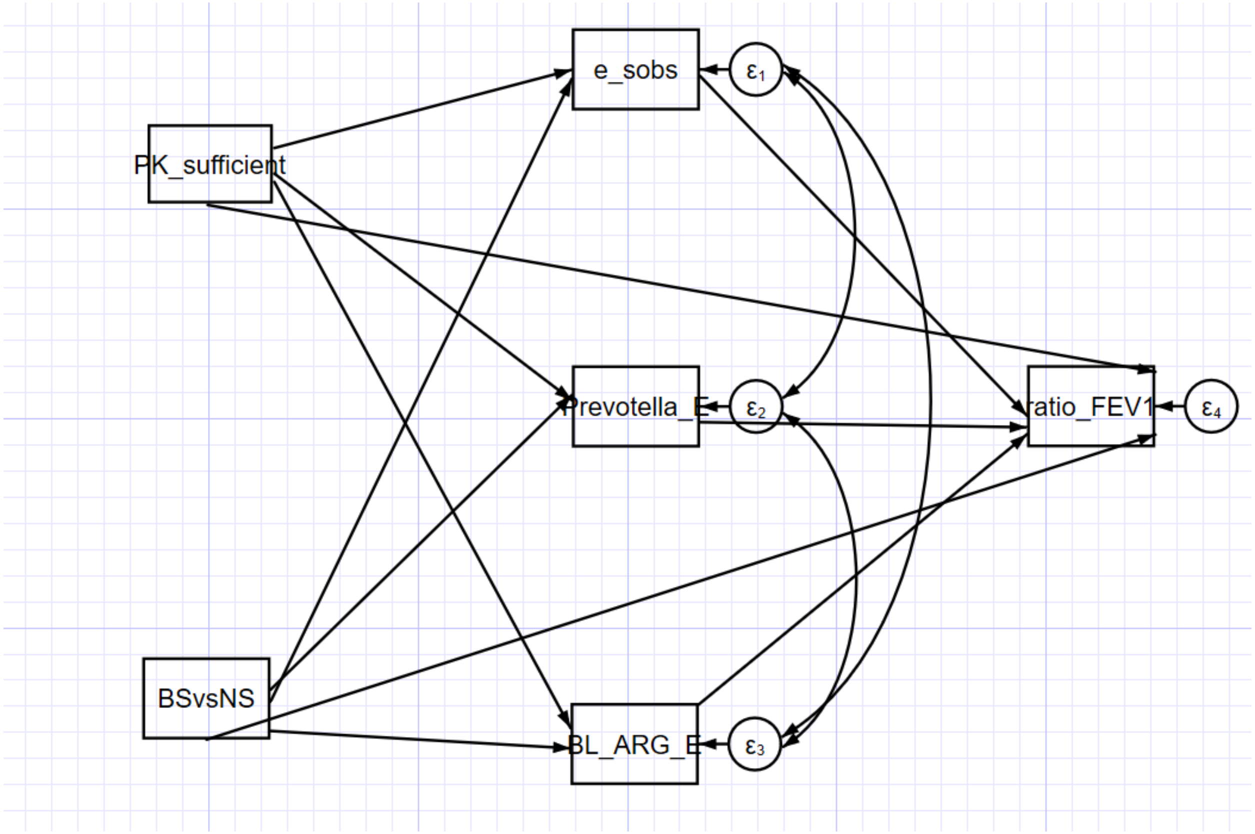

Supplemental Figure 1. Structural Equation Model. PK_sufficient, beta-lactam pharmacokinetic sufficient; BSvsNS, antibiotic spectrum; e_sobs, species observed at pulmonary exacerbation; Prevotella_E, relative abundance of Prevotella at pulmonary exacerbation; BL_ARG_E, relative abundance of beta-lactam antibiotic resistance genes at pulmonary exacerbation; ratio_FEV1, the ratio of the percent predicted forced expiratory volume in one second (ppFEV1) at the end of antibiotic treatment compared to the best ppFEV1 in the 6 months prior to the pulmonary exacerbation. In the multiple versions of the model ran, species observed was replaced by both Shannon diversity and the inverse Simpson index, while ratio_FEV1 was replaced by the improvement of ppFEV1 at end of treatment compared to the ppFEV1 at pulmonary exacerbation.

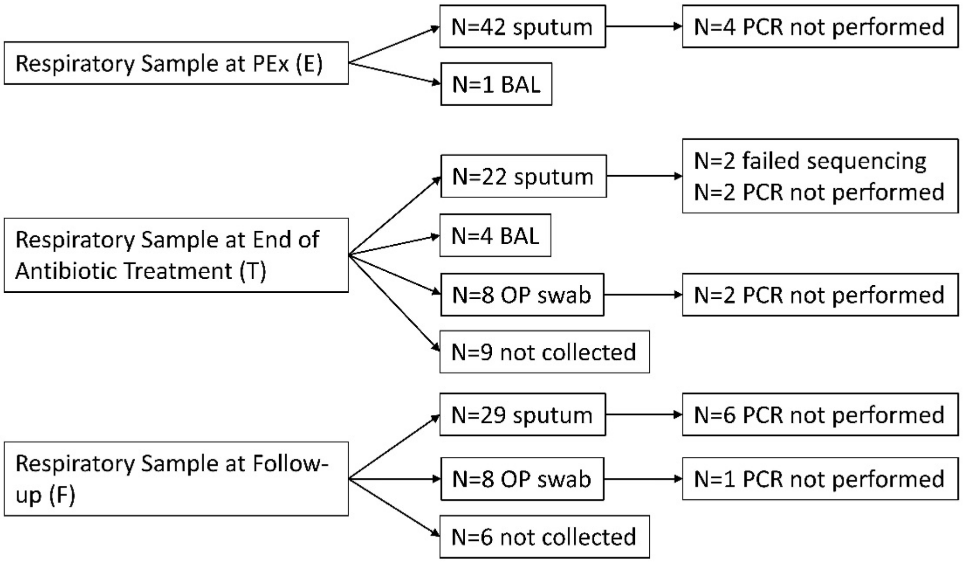

Supplemental Figure 2. Flow diagram of respiratory samples by time point. E, pulmonary exacerbation; T, end of antibiotic treatment; F, follow up; BAL, bronchoalveolar lavage; OP, oropharyngeal; PCR, polymerase chain reaction.

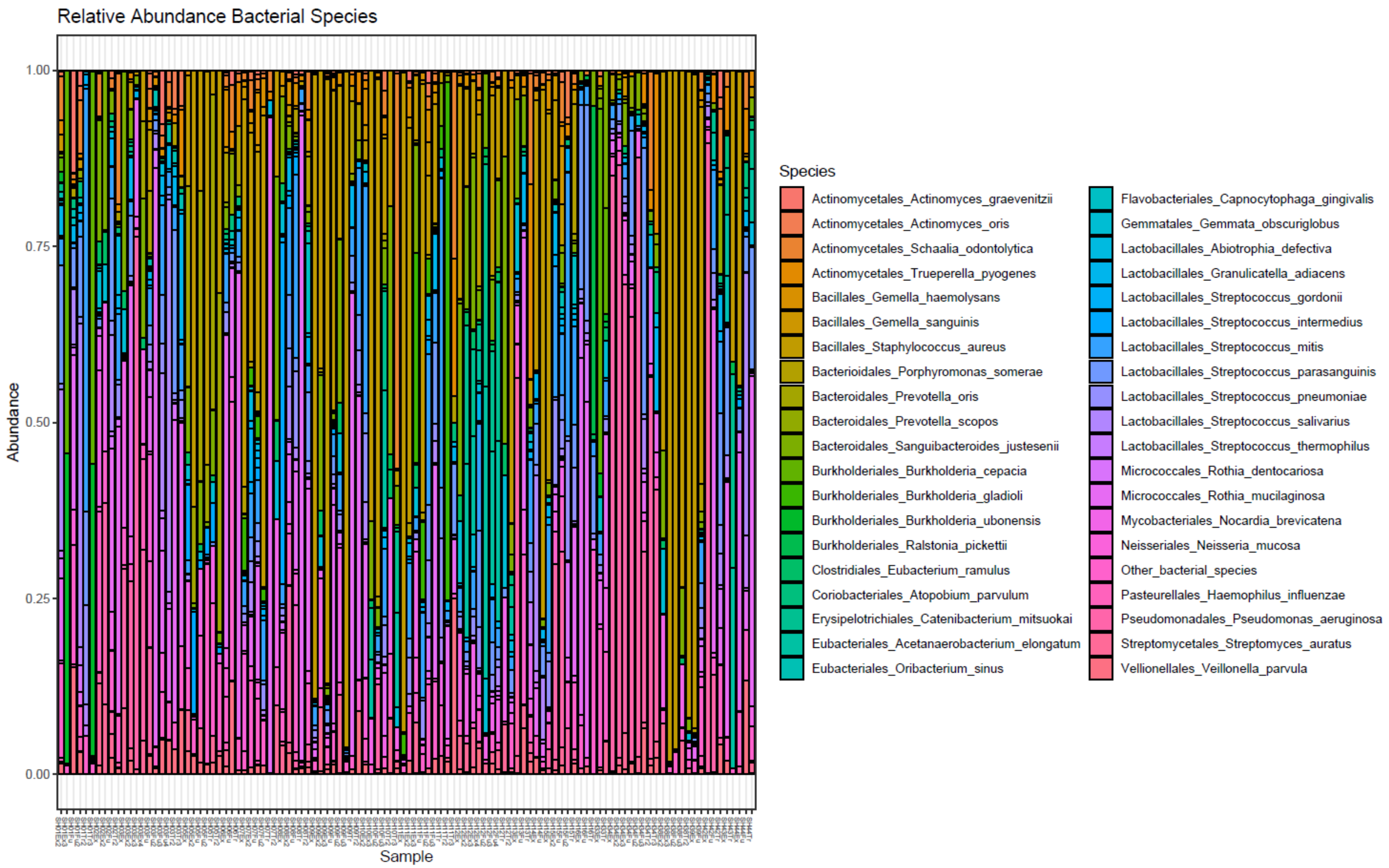

Supplemental Figure 3. Relative abundance of bacterial species by time point. The species were first filtered by those species that contributed to >5% relative abundance of an individual sample. Next, the species were ordered based on their contribution to the whole study cohort. This graph reports the top 39 most abundant bacterial species, with the contributions of the remaining 70 species included in the Other_bacterial_species category. The names of the bacteria are written as Order_Genus_Species.

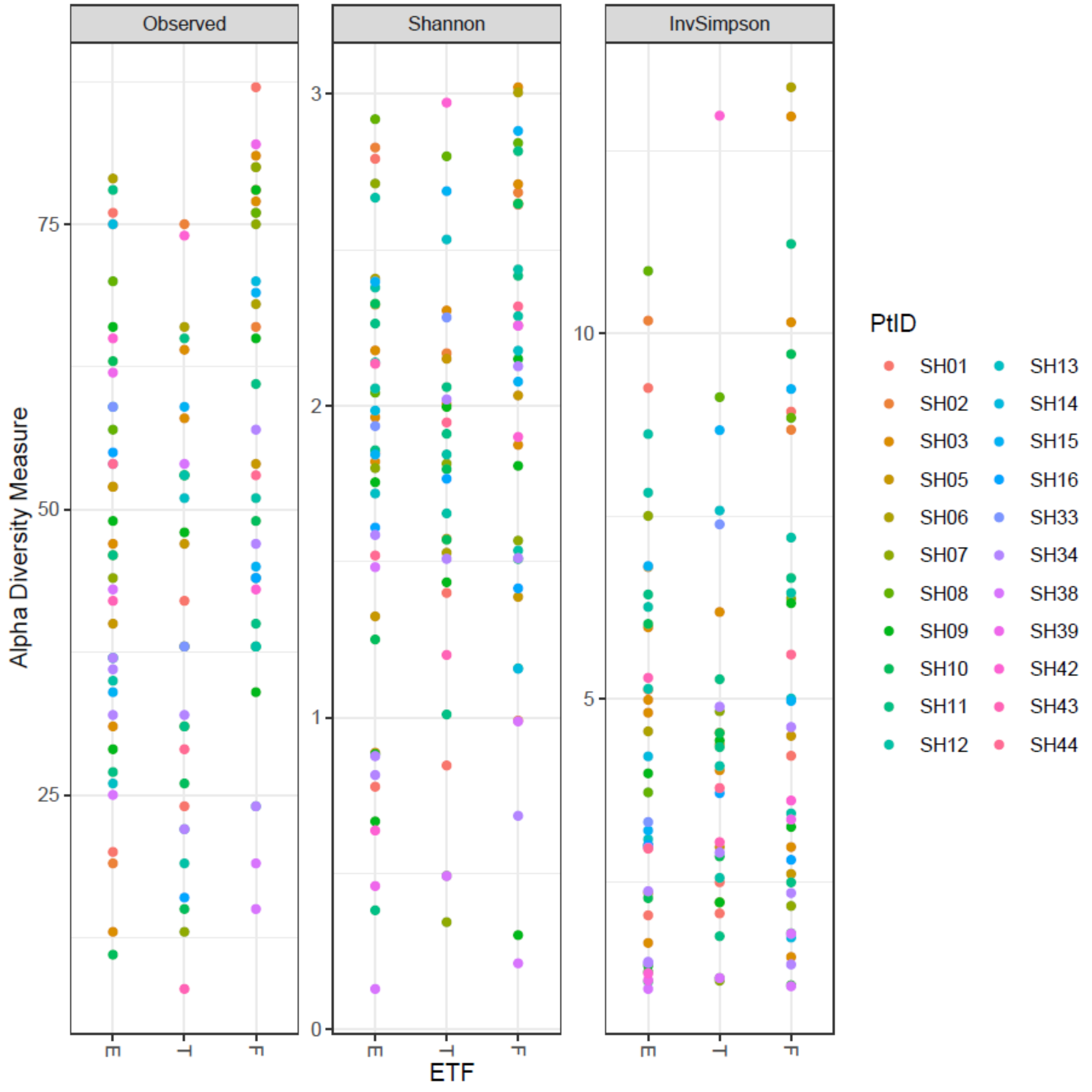

Supplemental Figure 4. Alpha diversity by time point. E, pulmonary exacerbation; T, end of antibiotic treatment; F, follow up.

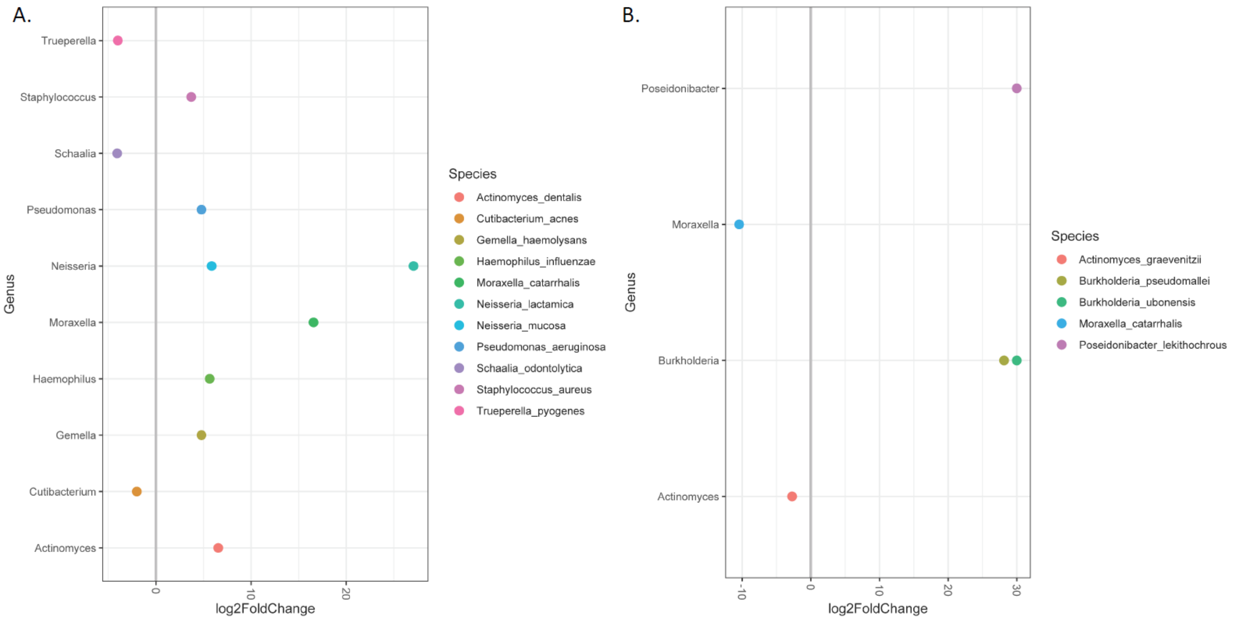

Supplemental Figure 5. Differential abundance of bacterial species by time point. Panel A. Differential abundance between pulmonary exacerbation and end of treatment. Panel B. Differential abundance between pulmonary exacerbation and follow-up. A positive log2 fold change was significantly higher in pulmonary exacerbation (right side), while a negative log2 fold change was significantly higher at the end of treatment (Panel A) or follow-up (Panel B). All adjusted p-values < 0.05.

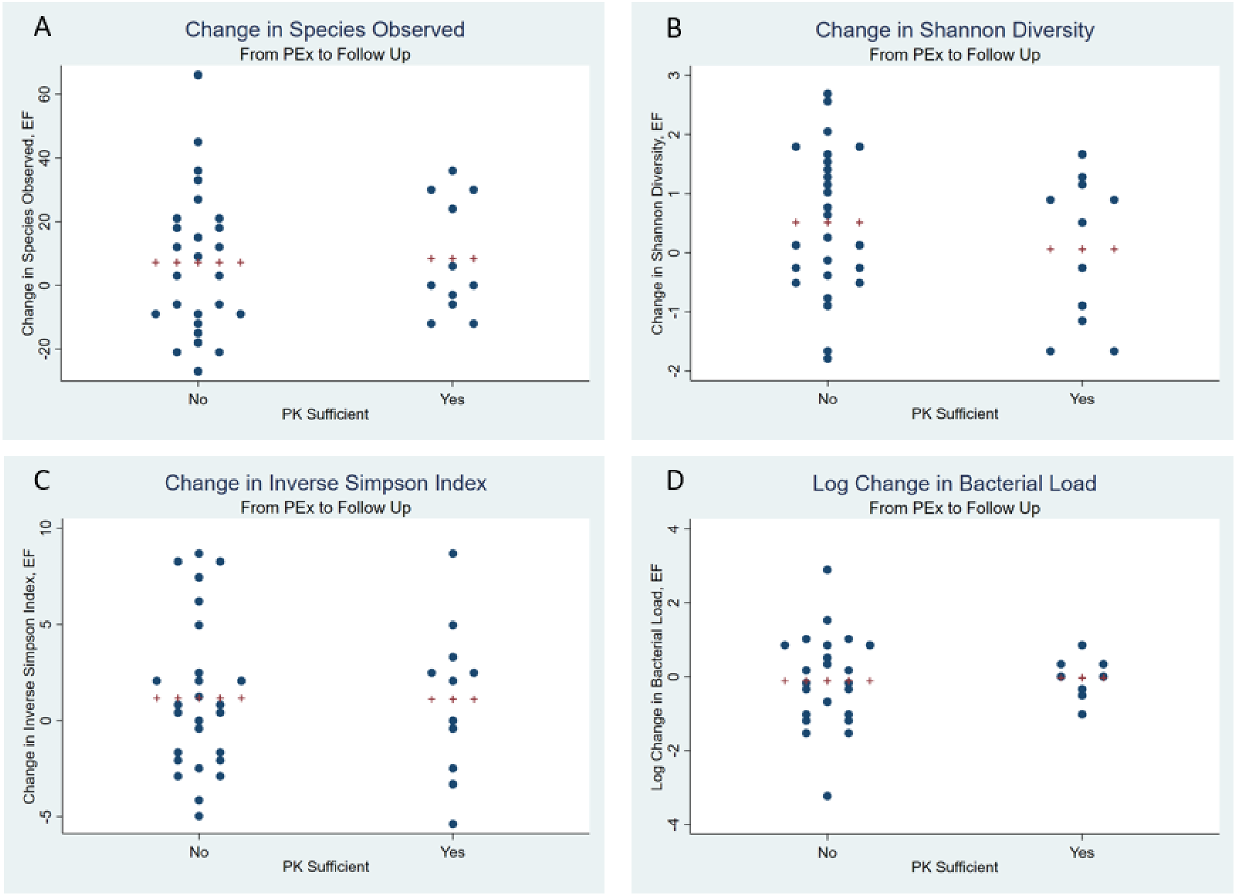

Supplemental Figure 6. Change in Alpha Diversity and Bacterial Load by Beta-Lactam Pharmacokinetic Exposure from Pulmonary Exacerbation to Follow Up. Panel A. Change in Species Observed. Panel B. Change in Shannon Diversity. Panel C. Change in Inverse Simpson Index. Panel D. Log Change in Bacterial Load. PK, pharmacokinetic; PEx, pulmonary exacerbation; E, pulmonary exacerbation; F, follow up; EF, change between pulmonary exacerbation and follow-up.

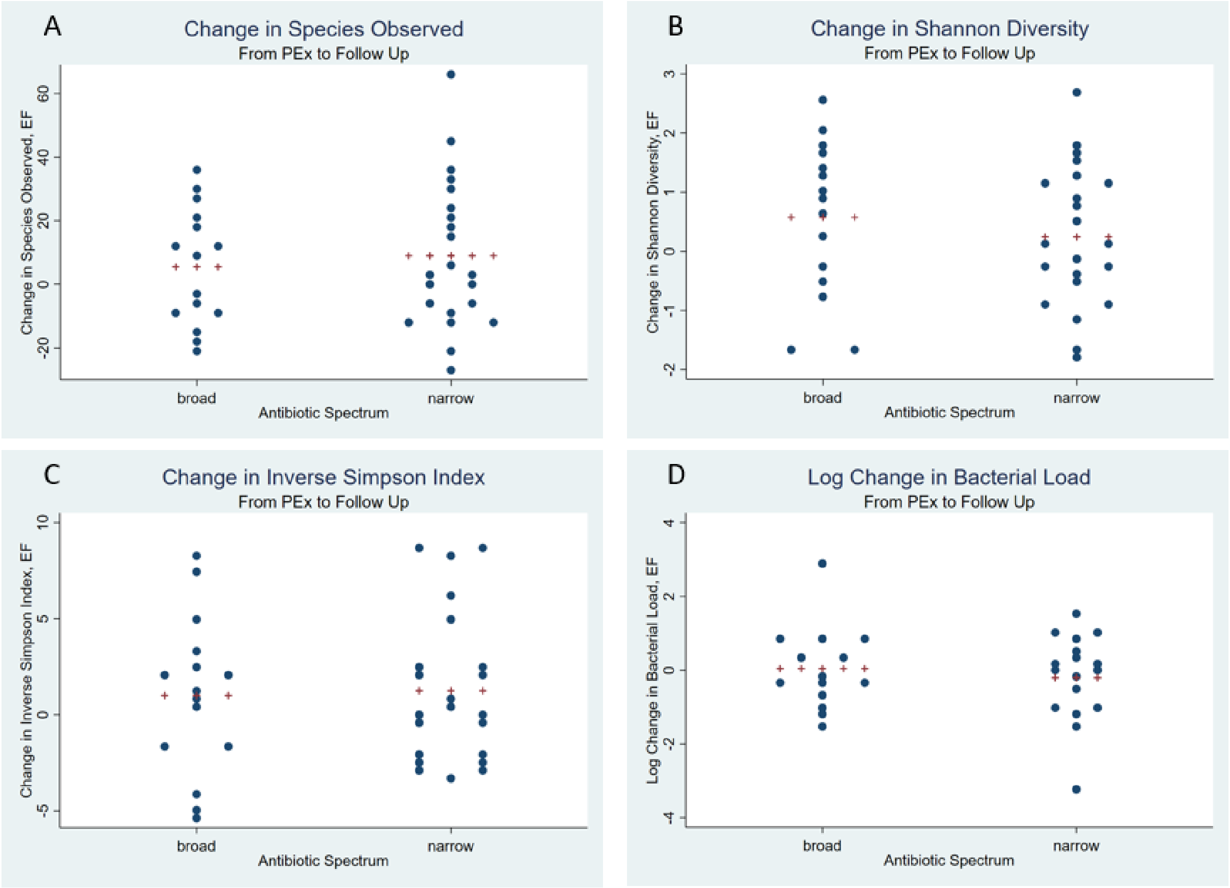

Supplemental Figure 7. Change in Alpha Diversity and Bacterial Load by Antibiotic Spectrum Exposure from Pulmonary Exacerbation to Follow Up. Panel A. Change in Species Observed. Panel B. Change in Shannon Diversity. Panel C. Change in Inverse Simpson Index. Panel D. Log Change in Bacterial Load. PEx, pulmonary exacerbation; E, pulmonary exacerbation; F, follow up; EF, change between pulmonary exacerbation and follow-up.

Supplemental References

1. Konstan, M. W. Characterizing aggressiveness and predicting future progression of CF lung disease. *J Cyst Fibros* **8 Suppl 1**, S15-9 (2009).
2. Fuchs, H. J. *et al.* Effect of aerosolized recombinant human DNase on exacerbations of respiratory symptoms and on pulmonary function in patients with cystic fibrosis. The Pulmozyme Study Group. *N. Engl. J. Med.* **331**, 637–42 (1994).
3. Cockcroft, D. W. & Gault, M. H. Prediction of creatinine clearance from serum creatinine. *Nephron* **16**, 31–41 (1976).
4. Soulsby, N., Greville, H., Coulthard, K. & Doecke, C. What is the best method for measuring renal function in adults and children with cystic fibrosis? *J. Cyst. Fibros.* **9**, 124–129 (2010).
5. Schwartz, G. J. *et al.* New equations to estimate GFR in children with CKD. *J. Am. Soc. Nephrol.* **20**, 629–637 (2009).
6. Downes, K. J., Hahn, A., Wiles, J., Courter, J. D. & Vinks, A. A. Dose optimisation of antibiotics in children: Application of pharmacokinetics/pharmacodynamics in paediatrics. *Int. J. Antimicrob. Agents* **43**, 223–230 (2014).
7. McIver, L. J. *et al.* BioBakery: A meta'omic analysis environment. *Bioinformatics* **34**, 1235–7 (2018).
8. Lakin, S. M. *et al.* MEGARes: An antimicrobial resistance database for high throughput sequencing. *Nucleic Acids Res.* **45**, D574-80 (2017).
9. Zemanick, E. T. *et al.* Inflammation and Airway Microbiota during Cystic Fibrosis Pulmonary Exacerbations. *PLoS One* **8**, e62917 (2013).
10. Nadkarni, M. A., Martin, F. E., Jacques, N. A. & Hunter, N. Determination of bacterial load by real-time PCR using a broad-range (universal) probe and primers set. *Microbiology* **148**, 257–266 (2002).
11. Iwahara, K. *et al.* Detection of cfxA and cfxA2, the β-lactamase genes of Prevotella spp., in clinical samples from dentoalveolar infection by real-time PCR. *J. Clin. Microbiol.* **44**, 172–176 (2006).
12. Handal, T., Olsen, I., Walker, C. B. & Caugant, D. A. Detection and characterization of β-lactamase genes in subgingival bacteria from patients with refractory periodontitis. *FEMS Microbiol. Lett.* **242**, 319–324 (2005).
13. Giraud-Morin, C., Madinier, I. & Fosse, T. Sequence analysis of cfxA2-like β-lactamases in Prevotella species. *J. Antimicrob. Chemother.* **51**, 1293–1296 (2003).
